# Retraction: Metabolomics reveals that PPARα activation protects against lithocholic acid-induced liver injury

**DOI:** 10.1039/d3ra90008h

**Published:** 2023-01-31

**Authors:** Qi Zhao, Rui Yang, Fang Liu, Jing Wang, Dan-Dan Hu, Xiu-Wei Yang, Fei Li

**Affiliations:** a State Key Laboratory of Phytochemistry and Plant Resources in West China, Kunming Institute of Botany, Chinese Academy of Sciences Kunming 650201 China lifeib@mail.kib.ac.cn +86-871-65216953; b University of Chinese Academy of Sciences Beijing 100049 China; c School of Pharmaceutical Science, Yunnan Key Laboratory of Pharmacology of Natural Products, Kunming Medical University Kunming 650500 China; d School of Pharmaceutical Sciences, Peking University Health Science Center, Peking University Beijing 100191 China

## Abstract

Retraction of ‘Metabolomics reveals that PPARα activation protects against lithocholic acid-induced liver injury’ by Qi Zhao *et al.*, *RSC Adv.*, 2017, **7**, 49849–49857, https://doi.org/10.1039/C7RA08823J.

The Royal Society of Chemistry, with the agreement of the named authors, hereby wholly retracts this *RSC Advances* article due to concerns with the reliability of the data in the published article. The authors confirmed that the western blot images in Fig. S3A had been inappropriately manipulated. Given the significance of the concerns about the validity of the data, the findings presented in this paper are no longer reliable.

Fang Liu, Jing Wang and Xiu-Wei Yang were contacted but did not respond.

Signed: Qi Zhao, Rui Yang, Dan-Dan Hu and Fei Li

Date: 23rd January 2023

Retraction endorsed by Laura Fisher, Executive Editor, *RSC Advances*

## Supplementary Material

